# Supplementary material for: Identification of photoperiod-induced specific miRNAs in the adrenal glands of Sunite sheep (Ovis aries)
Source: Front Vet Sci. 2022 Jul 22;9:888207. doi: 10.3389/fvets.2022.888207 (PMC9354845; doi:10.3389/fvets.2022.888207)
Supplement: Supplementary file 1 [file Data_Sheet_1.ZIP › Supplementary file/miRDeep2 file.zip!/miRDeep2 file/Novel_Structure/LP42A3/Novel_418.pdf]

Provisional ID	:	NC_019466.2_11780
Score total	:	0.9
Score for star read(s)	:	-1.3
Score for read counts	:	0
Score for mfe	:	0.6
Score for randfold	:	1.6
Score for cons. seed	:	
Total read count	:	45
Mature read count	:	45
Loop read count	:	0
Star read count	:	0

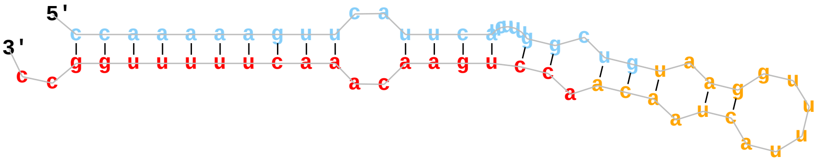

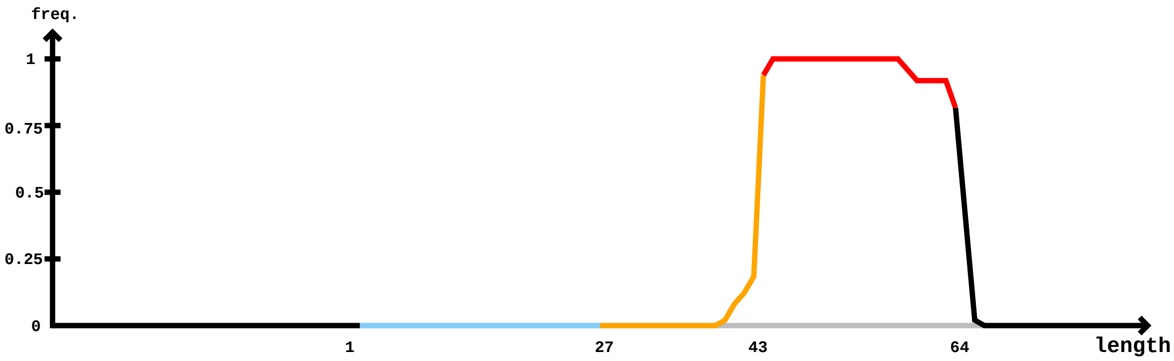


```
                                            Star                                          Mature
5'- aaucaaaaguauccauucuuacagguuuggccaaaaaguucauucaucuuuggcuguaagguuuuacuaacaaccugaacaaacuuuuuggccaacccaauaguucaucauc   -3'    exp
    ........(((........))).((.((((((((((((((..((((.....((.(((.((......)).))).))))))..)))))))))))))).))..............    reads      mm         sample
    ....................................................................aaAaaccugaacaaacuuuu........................    1          1          seq
    .....................................................................aAaaccugaacaaacuuu.........................    2          1          seq
    .....................................................................aAaaccugaacaaacuuuu........................    1          1          seq
    ......................................................................caaccugaacaaacuuuuuggc....................    2          0          seq
    .......................................................................aaUcugaacaaacuuuuuggc....................    1          1          seq
    .......................................................................aaUcugaacaaacuuuuuggcc...................    2          1          seq
    ........................................................................aAcugaacaaacuuuuuggc....................    1          1          seq
    ........................................................................accugaacaaacuuuuuggcc...................    1          0          seq
    ........................................................................aUcugaacaaacuuuuuggcc...................    34         1          seq
    ........................................................................aUcugaacaaacuuuuuggccaa.................    1          1          seq
    .........................................................................Ucugaacaaacuuuuuggc....................    1          1          seq
    .........................................................................Ucugaacaaacuuuuuggcca..................    2          1          seq
```
